# Magnetic resonance imaging vs. two- and three-dimensional computed tomography scans for assessment of glenoid inclination and version

**DOI:** 10.1016/j.jseint.2024.08.182

**Published:** 2024-08-28

**Authors:** Thomas Wittmann, Tim Rieger, Sandra Krawczyk, Tobias Helfen, Inês Santos, Patric Raiss

**Affiliations:** a Department of Orthopaedics and Trauma Surgery, Musculoskeletal University Center Munich (MUM), LMU University Hospital, LMU Munich, Munich, Germany; b Department for Shoulder Surgery, OCM Clinic Munich, Munich, Germany; c Arthrex GmbH, Munich, Germany

**Keywords:** Shoulder arthroplasty, Preoperative planning, MRI, CT, Glenoid assessment, Glenoid version, Glenoid inclination

## Abstract

**Background:**

Accurate glenoid component placement is crucial for anatomic (aTSA) or reverse (rTSA) total shoulder arthroplasty. Preoperative glenoid assessment in computed tomography (CT) scans with or without planning software seems to be the established method to plan implant positions. Magnetic resonance imaging (MRI) scans can also display the glenoid bone for preoperative assessment while reducing radiation exposure. Therefore, the objective of this study was to manually assess the glenoid version and inclination in 2D MRI and CT scans in cases with degenerative shoulder pathologies. The results were compared to those of an automated 3D planning software to validate the imaging modality for preoperative glenoid assessment.

**Methods:**

MRI and CT scans of 146 patients (n = 41 aTSA; n = 105 rTSA) were included in this retrospective, single-center study. Glenoid version and inclination were measured manually according to Friedman et al and Maurer et al on CT and MRI scans by two observers. Subsequently, the results were compared to the automated measurements performed by planning software. A repeated-measures analysis of variance was performed to compare the measured angles, and the interobserver and intraobserver reliability were calculated using the intraclass correlation coefficients. The level of significance was set at *P* < .05.

**Results:**

The average glenoid inclination measured in CT scans was 7.94° ± 7.33°, in MRI scans it was 8.56° ± 7.34°, and in automated planning software it was 7.87° ± 7.60°. The analysis of variance revealed significant differences in mean inclination between 2D MRI and 2D CT (*P* < .0005) and between MRI and automated software (*P* = .011). No significant difference was found between 2D CT scans and automated planning software (*P* = 1.000). The mean glenoid version measured in 2D CT scans was −7.94° ± 10.86°, in 2D MRI scans it was −8.04° ± 10.80°, and −8.32° ± 11.53° in the automated planning software. There was no significant difference in between measurement methods (*P* = .339). Interobserver reliability analysis showed no statistical differences between the two observers. All measurements had excellent intraobserver reliability.

**Conclusion:**

Preoperative assessment of glenoid version and inclination is crucial in ensuring precise implant positioning and orientation in aTSA and rTSA. This study observed a significant level of concordance between manual and automated measuring techniques utilizing MRI and CT scans. The mean glenoid inclination exhibited a statistically significant difference of less than 1° across the assessment modalities, and no difference for glenoid version was noted. It seems to be questionable if this finding is clinically relevant. MRI may serve as a viable and safe option for assessing glenoid morphology, version, and inclination if CT scans are not available.

The positioning of glenoid components may affect radiologic and clinical outcomes for both anatomic total shoulder arthroplasty (aTSA) and reverse total shoulder arthroplasty (rTSA).^7^^,^^13^^,^^24^, ^25^, ^26^ Superiorly placed or superiorly inclined components may be associated with scapular notching or component loosening.^7^^,^^13^^,^^24^, ^25^, ^26^ In cases with a challenging intraoperative exposure of the glenoid or substantial glenoid erosion, the risk of mispositioning of the glenoid component may increase. Therefore, preoperative evaluation of the glenoid bone stock, glenoid version, and inclination are crucial to achieve sufficient implant position and orientation.^3^^,^^12^^,^^15^ In addition to plain radiographs, glenoid morphology can be evaluated in two-dimensional (2D) and three-dimensional (3D) computed tomography (CT) scans, which offer a precise visualization of all features, including glenoid version and inclination.^3^^,^^29^^,^^30^ In recent years, 3D planning software has been developed, which uses preoperative CT imaging data to simulate implant positioning.^14^^,^^21^ These software tools are offering surgeons access to 3D scapula models, automated assessments of glenoid indices, and the ability to conduct component trials while evaluating potential bony impingement and analyzing the range of motion.^3^^,^^21^

However, the condition of the rotator cuff influences the final decision of shoulder arthroplasty type and can be assessed preoperatively by a clinical examination, sonography or magnetic resonance imaging (MRI).^10^ As MRI scans also depict the glenoid bone, it may be utilized for preoperative assessment in cases with degenerative shoulder conditions planned for arthroplasty, resulting in a reduction in the patient's exposure to radiation.^5^^,^^28^ But, it is unclear to what extent glenoid assessment carried out using MRI scans yields comparable results to well-established CT-based techniques or automated measures by planning software. Hence, the aim of this study was to manually assess glenoid version and inclination in standardized 2D MRI and CT scans in cases with degenerative shoulder diseases and to subsequently compare the findings with those of an automated 3D planning software. Our hypothesis was that there is no difference among the imaging modalities and methods.

## Materials and methods

This retrospective study evaluated demographic and imaging data obtained from an anonymous case series of one specialized shoulder center. Based on the guidelines established by the ethics committee of the Bavarian medical chamber, no specific institutional review board statement was necessary for this anonymous, retrospective study (IRB Nr. 2021-1148).

### Patients

A total number of n = 406 cases who underwent either aTSA or rTSA by one fellowship-trained shoulder surgeon between November 2017 and February 2022 in one specialized center (Munich, Germany) were screened for inclusion. Cases in which either a aTSA or rTSA was implanted due to (1) primary glenohumeral osteoarthritis or (2) cuff-tear-arthropathy, with (3) no prior surgical intervention on the affected shoulders, were included. In addition, complete (4) preoperative anteroposterior (a.p.) and axillary radiographs, an MRI scan, a CT scan, and a preoperative planning report had to be available in all included cases. All cases with (1) incomplete preoperative imaging or absence of preoperative planning, (2) other indications for joint replacement, (3) a history of glenoid fracture or other types of bony irregularities affecting the glenoid, (4) a prior revision surgery performed on the affected shoulder, (5) presence of infections, (6) acute fractures, (7) neurologic diseases, or (8) rheumatic diseases were excluded.

### Imaging protocol, preoperative planning and image analysis

A true a.p. and axillary radiograph, standardized MRI scans, and CT scans with the same slice thickness and sequences of the affected shoulders were obtained preoperatively and analyzed retrospectively in all cases. All MRI scans focused on the glenohumeral joint, with a various amount of the scapula body displayed. All MRI scans were performed using closed MRI scanners with the patient in a prone position. To ensure compatibility with the planning software, the CT scans were preoperatively conducted according to the recommended parameters provided by the manufacturer (Stryker, Kalamazoo, MI, USA). Every case underwent preoperative planning using Blueprint software (Stryker, Kalamazoo, MI, USA). The software offers a virtual 3D shoulder model based on the CT scan data with automatic measurements for glenoid version, inclination, and humeral head subluxation. In every case, automated measurements were recorded for the purpose of statistical analysis.

In cases with primary osteoarthritis, glenoid erosion was graded according to the classification by Walch et al in axial CT images.^1^^,^^29^ The grading system according to Favard et al was used to classify glenoid erosion in cases with cuff tear arthropathy based on true a.p. x-rays.^27^

**Figure 1:**
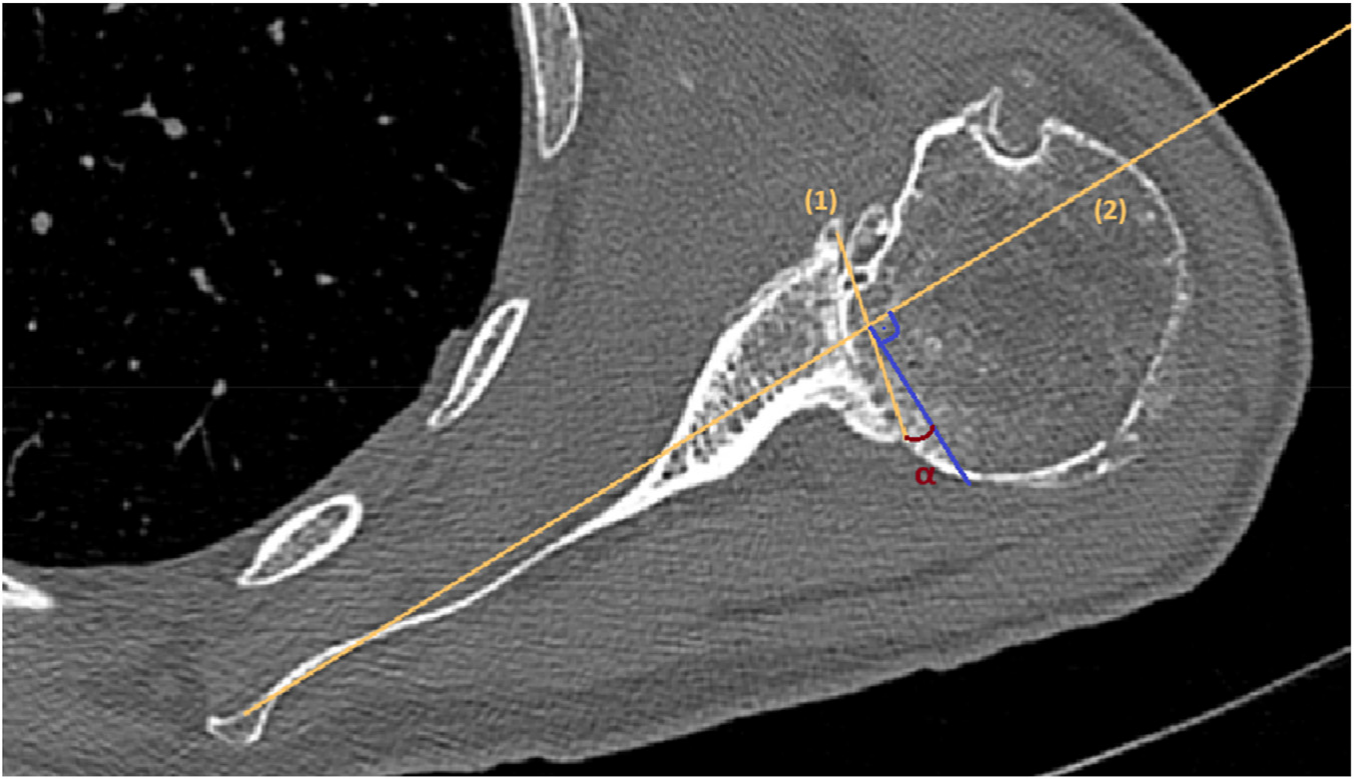
The glenoid version α according to Friedman et al is the angle between the scapular axis (1) and the intermediate glenoid joint line (2) in axial view one slice below the coracoid process.^9^

A manual assessment of glenoid version was conducted on axial view slices of CT according to Friedman et al and applied for measurements in MRI scans in the same manner.^9^ According to this method, the transversal scapular axis is determined by identifying the most medial point of the scapula and the central point of the glenoid fossa in the first axial slice beneath the coracoid process. Subsequently, the intermediate joint line was drawn to establish a connection between the foremost and hindmost portions of the glenoid, taking into account the presence of eccentric bone loss. Glenoid body osteophytes were not taken into account in this particular stage. The glenoid version refers to the angle α formed between the perpendicular line to the scapular axis (neutral) and the intermediate joint line. Retroversion is operationally defined as the posterior intermediate joint line being medial to the perpendicular line to the scapular axis and anteversion as the posterior intermediate joint line being lateral (Fig. 1). Retroversion was defined as negative version and anteversion as positive.

**Figure 2:**
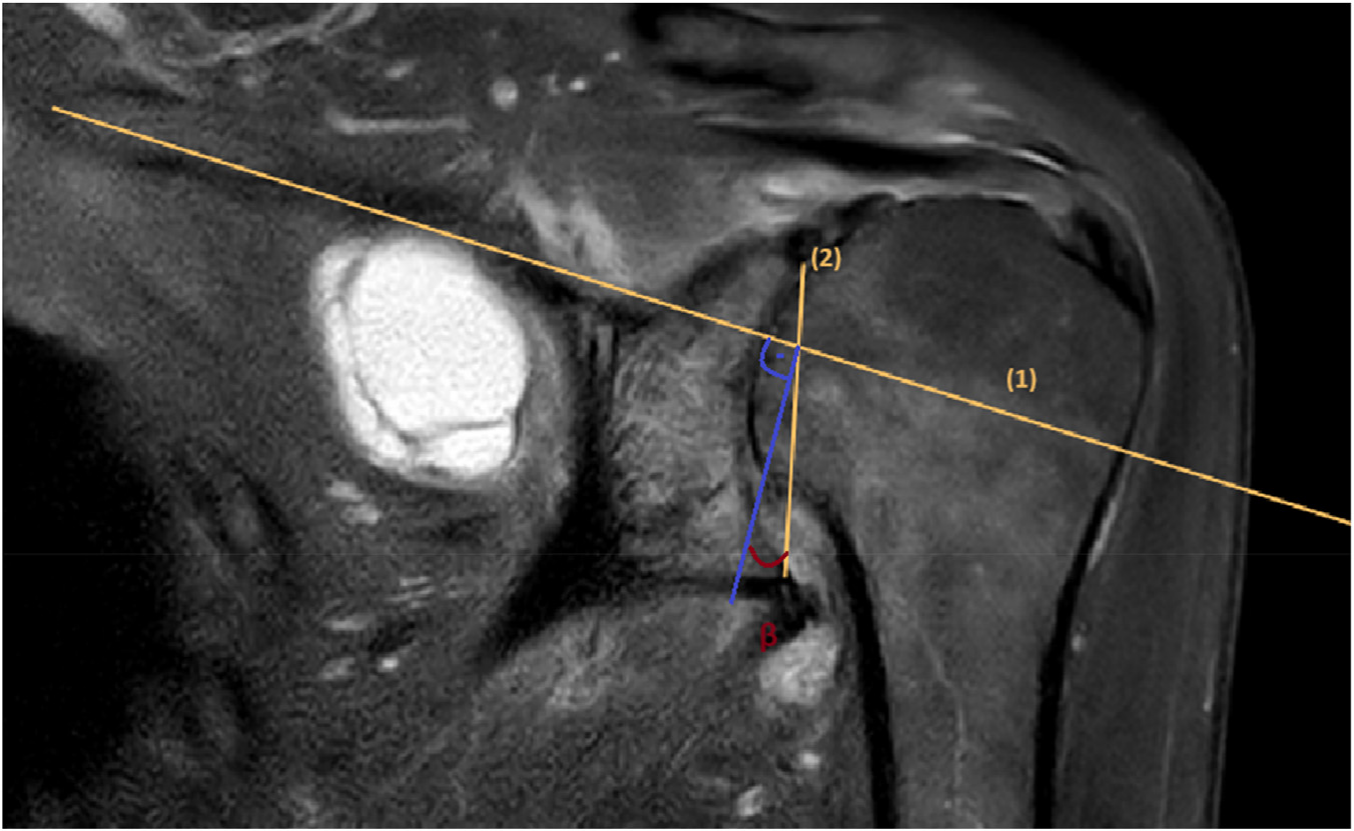
To measure glenoid inclination according to Maurer et al, the supraspinatus fossa line (1) and the glenoid fossa line (2) is drawn.^17^ The glenoid inclination β is the angle between a perpendicular line to the supraspinatus fossa line and the glenoid fossa line.

The assessment of glenoid inclination was conducted in the coronal view of CT scans and coronal T2-sequence in the MRI following the methodology established by Maurer et al.^17^ According to this method, the glenoid inclination is the angle β between a perpendicular line to the supraspinatus fossa line and the central glenoid fossa line, which connects the highest and lowest points of the glenoid cavity (glenoid inclination = 90°−β). (Fig. 2)

### Statistical analysis

The CT and MRI images were independently measured for each case by two observers who were blinded to each other's findings. One of the observers performed the measurements twice for the intraobserver analysis. A repeated-measures analysis of variance with a Greenhouse-Geisser correction was performed using SPSS Statistics software (version 26.0.0.1; IBM Corp., Armonk, NY, USA) to detect significant differences between the measured angles. The level of significance was set at 0.05 (*P* < .05). The reproducibility of measurements was verified by determining the intraclass correlation coefficients (ICCs).

## Results

A final cohort of 146 cases with n = 41 aTSA cases and n = 105 rTSA cases were included. A total of n = 33 cases (8.1%) were excluded due to varying indications. Additionally, n = 37 cases (9.1%) were excluded because of revision surgery or past surgical procedures, while n = 190 cases (46.7%) were excluded due to incomplete imaging data. Among the included cohort, 57.5% (n = 84) were diagnosed with primary osteoarthritis and glenoid morphology was classified as A1 in n = 27 cases (32.1%), A2 in n = 15 cases (17.8%), B1 in n = 18 cases (21.4%), B2 in n = 24 cases (28.6%), and C in n = 1 case (1.2%). Of the n = 62 cases (42.5%) with cuff-tear arthropathy, n = 42 cases (67.7%) were classified as E0, n = 14 cases (22.6%) as E1, n = 4 cases (6.5%) as E2, and n = 2 cases (3.2%) as E3. In the study cohort, n = 70 (48.0%) were male and n = 76 (52.1%) were female patients, with an average age of 72.1 ± 9.7 years (range, 30-91 years).

The average glenoid inclination measured on CT scans was 7.94° ± 7.33 (range, −19.1° to 23.6°), in MRI scans 8.56° ± 7.34° (range, 15.3°-23.6°), and in automated software measurement 7.87° ± 7.60° (range, 18.0°-24.0°). The statistical analysis determined that the mean inclination differed significantly between the measurement methods (F (1.529, 221.672) = 7.850, *P* = .002). A post hoc analysis with a Bonferroni adjustment was performed and revealed that the inclination angles obtained from the MRI were significantly different from both the CT angles (0.608 degrees, 95% CI: 0.265-0.952, *P* < .0005) and the planning software angles (−0.690 degrees, 95% CI: 0.124-1.257, *P* = .011). The CT measurements exhibited no statistically significant differences compared to the planning software measurements (0.082°; 95% CI: −0.364 to 0.528, *P* = 1.000).

The mean glenoid version measured in CT scans was −7.94° ± 10.86° (range, −42.1° to 31.8°) and in MRI scans, it was −8.04° ± 10.80° (range, −43.80° to 35.00°). The automated software measurement provided a mean glenoid version of −8.32° ± 11.53° (range, −40.00° to 33.00°). There was no statistically significant difference in the mean glenoid version values across all measurement methods (F (1.451, 210.450) = 1.029, *P* = .339).

Interobserver reliability showed no statistical differences between the two observers for manual CT and MRI measurements (ICC > 0.994). Intraobserver reliability was excellent (ICC > 0.993) for all measurements (Tables I and II).

**Table I:** Interobserver reliability.

	ICC
Inclination	
CT	0.994
MRI	0.998
Version	
CT	0.998
MRI	0.998

*CT*, computed tomography; *MRI*, magnetic resonance imaging.

**Table II:** Intraobserver reliability analysis.

	ICC
Inclination	
CT	0.993
MRI	0.995
Version	
CT	0.964
MRI	0.998

*CT*, computed tomography; *MRI*, magnetic resonance imaging.

## Discussion

The evaluation of glenoid morphology, version, and inclination is crucial for precise positioning of shoulder arthroplasty components, which can be evaluated manually in CT scans or with the assistance of automated planning software.^3^^,^^9^^,^^17^^,^^30^ This study assessed the concordance of manual and automated techniques for measuring glenoid version and inclination in CT and MRI scans and found a strong agreement between all imaging modalities for glenoid version. However, there were notable differences in the measurements of inclination between MRI and CT scans.

Central or eccentric glenoid bone loss is a common finding in cases with advanced primary osteoarthritis or cuff tear arthropathy and poses a surgical challenge for component positioning.^13^^,^^29^ Unaddressed bone defects contribute to alterations in the native inclination and version of the glenoid joint line, resulting in possible mispositioning of glenoid components.^9^^,^^17^ Previous studies have highlighted that misaligned or mispositioned components may have a higher risk of complications and loosening.^2^^,^^7^^,^^10^^,^^11^^,^^20^^,^^21^^,^^26^^,^^31^ Furthermore, an increased inclination of the glenoid contributes to a higher critical shoulder angle, which was associated with a higher risk of periprosthetic radiolucent lines and loosening after aTSA in previous studies.^10^^,^^31^ In rTSA, the correct placement of the baseplate with addressing version and inclination may significantly impact the clinical as well as the radiological outcome and the stability of the prosthesis.^10^^,^^11^ A superior tilted baseplate increases joint stresses, elevates the probability of joint dislocation, and encourages scapular notching along the lower part of the scapular neck.^2^^,^^20^^,^^24^

To validate MRI as an alternative imaging technique for glenoid assessment, the current study analyzed the glenoid indices using 2D manual methods adopted in both CT and MRI imaging. Subsequently, these measurements were compared with automated measurements conducted by the planning software. Despite the application of different imaging techniques, the measurement of glenoid version also exhibited a strong agreement within the methods and imaging modalities as reported by Boileau et al.^3^ This seems to be interesting as MRI sequences have no particular focus on detailed bone presentation. However, there was a significant variation in glenoid inclination between both 2D MRI- and CT-based assessments, with an average inclination difference in MRI scans of −0.690° compared to the planning software and 0.608° compared to the CT scans (*P* < .011). It is questionable if the difference between measurement methods is clinically relevant as it was less than 1°.

Given the frequent use of MRI scans before surgery, preoperatively evaluating the glenoid in those MRI scans has the potential to reduce radiation exposure compared to CT scans solely for evaluation of the bony structures, while offering further information about the surrounding soft tissue and tendons.^5^^,^^16^^,^^19^ To trust in measurements and planned components, the accuracy and validity of MRI-based measurements must be ensured.^5^^,^^16^^,^^19^ At the moment, some studies reported strong concordance between measurements acquired from MRI and CT scans in cases of glenohumeral osteoarthritis.^5^^,^^19^^,^^22^ Cagle et al investigated 25 cases with total shoulder arthroplasty and compared the preoperative glenoid morphology, glenoid version, and humeral head subluxation in CT and MRI imaging.^5^ The authors reported a high level of agreement when assessing glenoid morphology, glenoid version, and humeral head subluxation in MRI and CT images among the same observer. However, there was only moderate agreement among different observers.^5^ Based on their results, the authors concluded that preoperative assessment of the glenoid using MRI scans is a precise alternative imaging method compared to CT scans.^5^ Rosenthal et al published similar results in their study in which they compared the accuracy of 3D-MRI and 3D-CT techniques in evaluating glenoid version and inclination in 29 cases with primary osteoarthritis.^22^ The study found no significant differences in glenoid version or inclination between the imaging techniques. Therefore, the authors concluded that 3D CT scans remain the preferred method for preoperative glenoid evaluation, but 3D MRI imaging can provide equally accurate results.

MRI scans often focus on the glenohumeral joint and do not always display the whole scapular body unlike CT scans.^16^^,^^19^ Parada et al aimed to evaluate the accuracy of measuring glenoid version using MRI compared to CT scans in 32 patients with shoulder instability.^19^ The study group assessed the glenoid version and also measured the portrayed scapular body width in MRI compared to CT scans and found no significant difference for glenoid version between the two imaging modalities, even though the MRI scan only showed a mean percentage of 78.2% of the scapular body width. Although the authors highlighted a concern regarding lower measurement accuracy due to only a section of the scapular being seen on the MRI scans, only 5 cases with a limited portrayed scapular body width showed a variation of more than 5 degrees in glenoid version. Furthermore, no significant correlation was observed in these cases between the depicted scapular body width and the change in glenoid version. The reported findings are consistent with the results of our investigation, which discovered a strong agreement between measurements of the glenoid version in MRI and CT scans.^5^^,^^19^^,^^22^ However, none of the previous studies evaluated possible differences in glenoid inclination and compared the results of MRI-based measurements to an automated software. In our study, we found a significant difference in glenoid inclination between MRI- and CT-based measurements, which may be attributed to the use of 2D vs. 3D measurement methodologies or the availability of alternative MRI reconstructions portraying the whole scapula body.^5^^,^^16^^,^^19^^,^^22^

Soft tissues have a higher percentage of molecules with longer transverse relaxation times compared to tendons or cortical bone, which have shorter or ultrashort transverse relaxation times.^6^^,^^8^^,^^28^ As a result, soft tissues are more easily observable with standard MRI sequences that utilize long echo times.^6^^,^^8^^,^^28^ Consequently, it is difficult to detect the signal of tissues with short or ultra-short transverse relaxation times due to their rapidly declining signal, which then appears “black” with a poor contrast.^6^^,^^8^ Recently, several MRI imaging sequences have been developed to enhance the distinction between soft tissue and cortical bone by using MRI sequences with short-to-zero echo times.^4^^,^^8^^,^^18^^,^^23^^,^^28^ Previous investigations have shown that these ultra-short echo time sequences or zero echo time (ZTE) sequences are valuable for enhancing the bony contrast and are therefore helpful for glenoid assessment in MRI scans.^4^^,^^8^^,^^28^ The study conducted by Breighner et al aimed to assess the concordance between CT and ZTE MRI imaging in terms of glenoid morphology assessment as well as measurements of the glenoid indices in 34 cases.^4^ The authors performed an analysis on standard-of-care MRI scans, CT scans, and ZTE MRI scans and found a strong agreement between ZTE MRI and CT scans, with the ZTE MRI scans showing better visualization of bone characteristics compared to the conventional standard-of-care MRI imaging.^4^

Our study analyzed standardized CT scans and MRI images acquired from the same practices and hospitals, but the inclusion criteria did not mandate the use of any specific MRI sequences or the imaging of the whole scapula. Therefore, we analyzed T1 and T2 sequences commonly employed in regular clinical practice and no short-to-zero echo time sequences were available. The preoperatively acquired CT scans were standardized to match the recommendations for the planning software to ensure their compatibility. Nevertheless, we found a high concordance for glenoid version but a significant difference in glenoid inclination when comparing manual and automated measures in all imaging modalities was noticed. The observed discrepancy for glenoid inclination may be attributed to a measurement error arising from inadequate contrast between the glenoid bone stock and labrum in nonstandardized MRI imaging lacking ultra-short echo time or ZTE sequences. In addition, the assessment of glenoid inclination in MRI scans was solely conducted using a manual 2D method, which may include potential errors when determining the appropriate coronal slice.

This study has some limitations. The lack of a consistent protocol for image acquisition in MRI scans increases unpredictability and inconsistency in image quality and slice thickness, hence posing challenges to the precision of the investigation. Furthermore, this study was conducted exclusively within a single institution and focused solely on individuals with degenerative shoulder pathologies.

Nevertheless, the study's cohort of 146 cases with degenerative joint pathologies amplifies the study's significance and the validity of the findings. Moreover, the interobserver and intraobserver reliability was excellent, indicating a high degree of consistency in the measurements conducted by the two observers, despite significant disparities among the assessed imaging modalities for glenoid inclination.

## Conclusion

This study observed a significant level of concordance between manual and automated measuring techniques utilizing MRI and CT scans. The mean glenoid inclination exhibited a statistically significant difference of less than 1° across the assessment modalities. MRI may serve as a viable and safe option for assessing glenoid morphology, version, and inclination if CT scans are not available.

## Disclaimers:

Funding: No funding was disclosed by the authors.

Conflicts of interest: Inês Santos, MSc is an employee of Arthrex Inc. Patric Raiss, M.D. is a paid Consultant and receives research support from Arthrex Inc. The other authors, their immediate families, and any research foundation with which they are affiliated have not received any financial payments or other benefits from any commercial entity related to the subject of this article.
